# Gratitude questionnaire: psychometric properties in Portuguese adult samples and evidence of validity

**DOI:** 10.1186/s41155-026-00396-z

**Published:** 2026-05-20

**Authors:** Félix Neto, Lourdes Rey, María Teresa Chamizo-Nieto, Agustín Wallace

**Affiliations:** 1 Faculty of Psychology and Educational Sciences, University of Porto, Porto, Portugal; 2 Faculty of Psychology and Speech Therapy, University of Malaga, Ampliación de Teatinos, Málaga, 29010 Spain

**Keywords:** Gender, Gratitude, Portuguese validity, Psychometrics

## Abstract

**Background:**

Gratitude has been increasingly studied as a key psychological strength associated with subjective well-being, positive interpersonal relationships, and mental health. The assessment of gratitude through validated instruments is essential for both research and clinical practice. Although the Gratitude Questionnaire 6 items (GQ-6) is widely used in various cultural contexts, there is a need to evaluate its psychometric properties and cultural relevance in Portuguese-speaking populations, particularly among adults.

**Objective:**

The present study aimed to examine the psychometric properties and validity evidence of the Portuguese version of the Gratitude Questionnaire, with a focus on its internal structure, score reliability, and evidence based on relations to other variables.

**Methods:**

A final sample of 810 Portuguese adults (Mage = 43.79; SD = 23.69) completed the Portuguese version of the GQ-6. Exploratory factor analysis and confirmatory factor analysis were conducted to evaluate the instrument’s internal structure. A subsample of 362 adults completed the revised 5-item version (GQ-5), along with measures of life satisfaction and loneliness, to assess evidence based on relations to other variables.

**Results:**

Findings supported a one-factor structure, with the 5-item version showing a better model fit than the original 6-item scale. The GQ-5 demonstrated significant positive correlations with life satisfaction and negative correlations with loneliness, supporting this source of validity evidence. The instrument also exhibited adequate internal consistency.

**Conclusion:**

The Portuguese version of the GQ-5 is a psychometrically sound and culturally appropriate measure for assessing gratitude in adult populations.

## Introduction

Throughout adulthood, people must face and adapt to various life challenges and changes in different areas (e.g., work, family, social or personal). The decisions people make and how they deal with these changes will have an impact on their health and well-being (e.g., Mehta et al., 2020). From Positive Psychology, there has been growing interest in identifying the psychological resources and strengths that individuals can develop to better cope with life challenges and promote health and well-being (e.g., Seligman & Csikszentmihalyi, 2000; Waters et al., 2022). In this framework, examining specific strengths and ensuring their adequate assessment becomes essential for advancing both research and applied interventions. Therefore, the present study aimed to examine the psychometric properties and validity evidence of the Portuguese version of the Gratitude Questionnaire (GQ-6; McCullough et al., 2002).

### Gratitude

One of the strengths that has seen an increase in research development and interest during the last decade has been gratitude (e.g., Kong et al., 2017; Waters et al., 2022). In the scientific setting, researchers have suggested several proposals to define gratitude. Thus, one can find from the consideration of gratitude as an affective trait proposed by McCullough et al. (2002) to its conceptualization as a lifestyle or vital orientation suggested by Wood et al. (2010). So, trying to agglutinate the different approaches, Portocarrero et al. (2020) defined the dispositional gratitude as “generalized tendency to respond with grateful emotion, by noticing and appreciating one’s positive experiences and achievements” (Portocarrero et al., 2020, p. 3).

A large body of research has analysed the benefits and relationships of gratitude on mental health and several psychological and social adjustment indicators (e.g., Jans-Beken et al., 2020; Portocarrero et al., 2020; Waters et al., 2022). Regarding mental health and psychological adjustment, numerous studies provide evidence on how gratitude can foster and increase the levels of life satisfaction, flourishing, happiness, positive affect, and well-being (e.g., Jafari, 2020; Kong et al., 2021; Portocarrero et al., 2020; Yang et al., 2021). In addition, its development help to prevent or diminish mental health problems, such as depression and anxiety symptomatology, perceived stress, or negative affect (e.g., Portocarrero et al., 2020; Yang et al., 2021; Yildirim & Alanazi, 2018). In relation to the benefits of gratitude on social adjustment, the scientific literature shows that gratitude not only promotes more positive and proactive social exchanges, reducing antisocial behaviours (Sasaki et al., 2020; Stieger et al., 2020), but also promotes and fosters more positive, closer, and more satisfying social relationships (e.g., Dennis & Ogden, 2022; Jiang et al., 2022; Wang, 2020). This facilitates social support and helps to reduce feelings of loneliness (e.g., Chui & Diehl, 2021; Frinking et al., 2020; Kong et al., 2021; You et al., 2022).

### Measurement of gratitude

To measure gratitude, some lists, scales and questionnaires have been developed, such as Gratitude Resentment and Appreciation Scale (GRAT; Watkins et al., 2003), Gratitude Adjective Checklist (GAC; McCullough et al., 2002), Transpersonal Gratitude Scale (Hlava et al., 2014), Gratitude Questionnaire – 6 items (GQ-6; Emmons & McCullough, 2003), Gratitude Questionnaire – 20 items (G-20; Bernabé-Valero et al., 2014), and Multi-Component Gratitude Measurement (MCGM; Morgan et al., 2017), among others.

Since there are various instruments to assess gratitude, Card (2019) carried out a meta-analysis of common measures. Its findings showed that GQ-6 was the most widely used measure of gratitude across a variety of contexts, countries, and settings (Card, 2019). The GQ-6 is a short, brief questionnaire developed by McCullough et al. (2002). It measures dispositional gratitude, including each of the facets of which it is composed: intensity, frequency, span, and density. Thus, a person with a high gratitude disposition will have feelings of gratitude more often and more intensely, and to a greater number of circumstances and people (McCullough et al., 2002). Initially, McCullough et al. (2002) proposed a version of 39 items, but after some studies and analyses performed the number of items was reduced. The final version of this instrument comprised 6 items, which measure a one-factor structure of gratitude. Moreover, the GQ-6 instrument showed adequate evidence based on relations to other variables, discriminant validity evidence, and high internal consistency of scores (McCullough et al., 2002). Furthermore, it offers several advantages. On the one hand, GQ-6 is a brief and short measure with adequate psychometric properties, which is why several researchers (e.g., Bernabé Valero et al., 2013; Dixit & Sinha, 2021; Gouveia et al., 2021) have considered it an ideal and useful tool to include in studies where several questionnaires and scales need to be used. On the other hand, GQ-6 have been adapted to different languages (e.g., Spanish, Italian, Chinese, Greek, Romanian, among others) and populations (e.g., children, adolescents, undergraduate or adults), obtaining adequate psychometric properties (e.g., Balgiu, 2020; Di Fabio, 2022; Ling et al., 2021; Magallares et al., 2018; Michailidis & Maridaki-Kassotaki, 2020; Rey et al., 2018). In particular, the GQ-6 has already evidenced score reliability and validity evidence using the Brazilian Portuguese (Gouveia et al., 2021; Souza et al., 2024).

A previous study with Portuguese college students provided initial evidence of validity and score reliability for a European Portuguese version of the GQ-6 (Neto, 2007), showing the expected associations with personality traits (i.e., agreeableness, extraversion, conscientiousness, openness to experience) and forgiveness. However, this work did not examine the factorial structure of the instrument, an essential aspect of the validation process. The present research therefore extends Neto’s contribution by evaluating the underlying factor structure of the GQ in European Portuguese and offering a more comprehensive psychometric assessment of the scale.

Various factors motivate different adaptations of this gratitude questionnaire, including cultural context, population, and sex differences. On the one hand, previous research has adapted a gratitude questionnaire to various population and cultural contexts to measure gratitude, based on the notion that gratitude is a culturally based expression (Garg et al., 2021) regardless of the age of the population. For example, in a comprehensive study, Tudge et al. (2016) investigated gratitude among children from different cultural backgrounds, including American, Brazilian, Chinese, and Russian. In this study the authors suggest that gratitude may have a common core with culturally universal characteristics, as well as socially constructed components that vary depending on the culture being studied. In adult samples, recent theoretical and empirical work suggests that the concept and experience of gratitude may encompass both culturally universal and culturally specific components. Morgan et al. (2014) argue that certain aspects of gratitude—such as its positive emotional core—appear to be relatively consistent across cultural contexts. At the same time, their findings indicate that other facets may vary meaningfully between cultures, including the emergence of negative elements within the experience of gratitude and the extent to which gratitude is shaped by culture-specific social norms. However, to the best of our knowledge, no empirical studies conducted in Portugal have examined whether such distinctions are present in Portuguese samples or how gratitude is conceptualised and expressed in this cultural setting. This gap in the literature underscores the relevance of developing and validating a Portuguese adaptation of the GQ. Establishing a psychometrically robust version of the instrument is essential not only for ensuring accurate assessment but also for enabling future research to explore potential culturally specific features of gratitude and to advance the understanding of this construct within the Portuguese context. For this reason, due to the culture-specific nature of gratitude, it is necessary to validate questionnaires measuring gratitude in various cultural contexts.

On the other hand, as noted by several authors (e.g., Bernabé Valero et al., 2013; Carmona-Halty et al., 2015) the GQ-6 questionnaire may exhibit psychometric properties variations depending on the population in which it is used. Consequently, different validations of the GQ-6 questionnaire have been conducted with samples of university students and adults from various countries. These validations have shown that the GQ-6 questionnaire possesses favourable psychometric properties for use with Chilean, Italian, Dutch and Chinese adult samples (Carmona-Halty et al., 2015; Di Fabio, 2022; Fuochi et al., 2018; Jans-Beken et al., 2015; Kong et al., 2021; Langer et al., 2016) and Brazilian, Indian, Filipino and Japanese university student samples (Garg et al., 2021; Gouveia et al., 2021; Llenares & Almeda, 2021; Sumi, 2017). In contrast, the GQ-5 questionnaire—an adaptation in which item 6 from the original instrument (“Long amounts of time can go by before I feel grateful to something or someone”) is removed—has demonstrated superior psychometric performance compared to the GQ-6 in university student samples from Spain, Romania, Taiwan and Turkey (Balgiu, 2020; Bernabé Valero et al., 2013; Chen et al., 2009; Yüksel & Oğuz Duran, 2012) as well as in adult samples from India, Ecuador and Spain (Cabrera-Vélez et al., 2019; Dixit & Sinha, 2021; Magallares et al., 2018). As several authors have suggested (e.g., Cabrera-Vélez et al., 2019; Chen et al., 2009; Dixit & Sinha, 2021), item 6 may exhibit lower factor loadings due to its greater semantic complexity. Plausible explanations include its higher level of abstraction, the presence of a temporal definition of gratitude that may hinder comprehension, and the multiple interpretations individuals may derive from its reversed wording (Cabrera-Vélez et al., 2019; Chen et al., 2009; Dixit & Sinha, 2021). To date, however, the psychometric functioning of the GQ-6 remains unknown in Portuguese adult populations. For this reason, this study tries to analyse this questionnaire in Portuguese samples. According to Card (2019), this helps in conducting comparative and cross-cultural studies, as well as drawing stronger conclusions.

### Gratitude and sex differences

Another important issue between researchers is, too, to examine the invariance across sex to ensure that any sex differences do not affect the interpretation of the GQ-6 questionnaire by both male and female participants. Previous studies have compared males and females in the GQ scores and the results are mixed. Thus, some studies found invariance across sex in this questionnaire (e.g., Cabrera-Vélez et al., 2019; Garg et al., 2021; Hudecek et al., 2020; Kong et al., 2017; Llenares & Almeda, 2021). In contrast, other findings showed that the GQ-6 worked differently for males and females (e.g., Dixit & Sinha, 2021; Gouveia et al., 2021). For this reason, analysing the invariance across sex would facilitate the comparison of gratitude scores between males and females, utilising the GQ-6 questionnaire.

### The present study

The validation of the GQ-6 in Portuguese samples offers several important benefits. It enhances diagnostic accuracy by reducing interpretation errors associated with unreliable measurements and improves the test’s sensitivity, thereby strengthening its ability to detect meaningful differences or changes in dispositional gratitude. A robustly validated instrument will also ensure comparability across groups, enabling conclusions based on genuine score differences rather than measurement artefacts. Furthermore, given that Portuguese is an official language in ten countries, this version not only advances research within Portugal but also complements the existing Brazilian adaptation, expanding opportunities for comparative and cross-cultural studies across Portuguese-speaking populations.

To address these gaps and strengthen the psychometric assessment of the GQ-6 in Portuguese adult samples, the present study evaluates the adaptability of the GQ-6 to the Portuguese context through two objectives. In our first aim, we examine the evidence of factorial structure and reliability of the GQ-6 scores using exploratory factor analysis, confirmatory factor analysis, invariance analysis across sex, and reliability estimates. Our hypothesis is that the one-factor model would exhibit good fit with the collected data. Ensuring measurement invariance across distinct groups, such as males and females, is crucial for performing meaningful group comparisons. Our expectation is that the measurement properties of the test are independent of the characteristics of the groups. Lastly, in terms of reliability, we anticipate an adequate internal consistency of the scores. In our second aim, we analyse the evidence based on relations to other variables for the scores derived from the instrument developed in the first objective within the Portuguese population. For this, we selected life satisfaction and loneliness as constructs due to their relationship with gratitude as evidenced in previous studies (e.g., Caputo, 2015; Chui & Diehl, 2021; Frinking et al., 2020; Portocarrero et al., 2020; Xiang & Yuan, 2021). The hypothesis was that gratitude would be significant and positively associated with life satisfaction and significant and negatively associated with loneliness.

## Method

### Participants

The psychometric properties of the GQ-6 were assessed in a sample of Portuguese adults (*N* = 812). Nevertheless, the data from two participants were removed because they had incomplete data. Therefore, the final sample consisted of 810 participants, of which 65.68% (532 participants) were women, 34.32% (278 participants) were men. The average age was 43.79 years (SD = 23.69) and 23.71% of them had primary education, 41.78% secondary education and 34.51% higher education. In relation to marital status, 51.67% were single, 28.50% were married, 7.81% were in a common-law relationship and 12.02% were other (divorced, widowed).﻿

This initial sample was divided into two samples using Solomon method (Lorenzo-Seva, 2022). The first sample consisted of 405 participants, of whom 65.68% (266 participants) were women and 34.21% (139 participants) were men. The average age was 43.78 years (SD = 23.71) and 23.43% of them had primary education, 42.57% secondary education and 34.01% higher education. In relation to marital status, 52.48% were single, 27.23% were married, 6.93% were in a common-law relationship and 13.37% were other (divorced, widowed). The second sample consisted of 405 participants, of whom 65.68% (266 participants) were women and 34.21% (139 participants) were men. The average age was 43.79 years (SD = 23.70) and 24.00% of them had primary education, 41.00% secondary education and 35.00% higher education. In relation to marital status, 50.87% were single, 29.78% were married, 8.68% were in a common-law relationship and 10.67% were other (divorced, widowed).

To examine the evidence based on relations to other variables of the Gratitude Questionnaire, a subsample of total sample (*n* = 362) completed additional measures assessing satisfaction with life and loneliness. In this subsample a 67.68% (245 participants) were women and 31.77% (115 participants) were men. The average age was 36.47 years (SD = 17.91), and 4.70% of them had primary education, 29.83% secondary education and 65.47% higher education. In relation to marital status, 56.08% were single, 22.93% were married, 14.36% were in a common-law relationship and 6.63% were other (divorced, widowed).

### Measures

Participants reported demographics, such as their age, sex, education level, marital status, and nationality. In addition, the following measures was used:

*Gratitude Questionnaire (GQ-6;* McCullough et al., 2002). This scale comprises six items such as, “If I had to list everything that I felt grateful for, it would be a very long list” (see Table 1). Response choices ranged from 1 (*Strongly disagree)* to 5 (*Strongly agree*). The third and sixth items were scored in reverse order, so greater values denote greater levels of gratitude. This instrument showed excellent score reliability and validity (α =.82; McCullough et al., 2002). The Portuguese version of the GQ-6 used was the one developed by Neto (2007) (α =.75). In that study the original English version of the GQ-6 was translated from English into Portuguese and then back to English using the guidelines proposed by Brislin (2000) for a back-translation method.

**Table 1:** Factor loadings from the exploratory factor analysis with the tested models (*N* = 405)

Item	6 item model factor loadings	5 item model factor loadings
1. I have so much in life to be thankful for [Tenho muito que agradecer na vida]	.81	.82
2. If I had to list everything that I felt grateful for, it would be a very long list [Elaboraria uma longa lista se tivesse que registar tudo pelo que estou agradecido(a)]	.79	.81
3. When I look at the world, I don’t see much to be grateful for [Quando olho para o mundo não vejo razões para exprimir muita gratidão]	.43	.40
4. I am grateful to a wide variety of people [Estou agradecido(a) a muitas pessoas]	.70	.70
5. As I get older I find myself more able to appreciate the people, events, and situations that have been part of my life history [À medida que fico mais velho(a) sou mais capaz de apreciar as pessoas, acontecimentos e situações que fizeram parte da minha história de vida]	.43	.43
6. Long amounts of time can go by before I feel grateful to something or someone [Eu demoro muito até me sentir grato a alguma coisa ou a alguém.]	.23	

*Satisfaction with Life Scale (SWLS;* Diener et al., 1985)*.* This scale evaluates the cognitive component of subjective well-being and comprises five items, such as: “If I could live my life over, I would change almost nothing”. The answers are evaluated on a seven-point Likert scale from 1 *(Strongly disagree)* to 7 *(Strongly agree)*, which higher scores indicating greater satisfaction with life. The Portuguese version showed excellent score reliability and validity in previous studies (Neto et al., 1990, α =.78; Neto, 1993, α =.78; Jovanović et al., 2022, α =.81). In the present study, Cronbach's alpha was.84.

*Loneliness (ULS-6*). This is a short form of the Revised UCLA Loneliness Scale (Russell et al., 1980). The ULS-6 consists of 6 statements (e.g., “People are around me but not with me”). Responses choices ranged from 1 (“*Never”*) to 4 (“*Often”*). Larger scores denote greater loneliness. The Portuguese version showed excellent score reliability and validity in previous studies (Neto, 1992, α =.82; Neto, 2014, α =.90). In this research, Cronbach’s α was.82.

### Procedure

The sample was recruited from community in public places by three trained research assistants (e.g., coffee bars, gardens, and railway stations) of Porto. The survey was administered to the participants using a standard paper and pencil format. The work was performed in accordance with the Declaration of Helsinki (2013), and local ethical norms. Respondents were informed on the goal of the work and gave informed consent. The anonymity of the respondents was reassured. Respondents were free to withdraw from the questionnaire anytime without giving any reason. Participants were unpaid volunteers.

### Data analysis

First, we made an exploratory factor analysis that shows a unifactorial structure and later we tested by confirmatory factor analysis (the sample was divided using the Solomon method (Lorenzo-Seva, 2022) to obtain two equivalent subsamples). In addition, we examined whether the gratitude scores present total invariance. Total variance means that the GQ scores are grouped into the same number of factors, with the same factor loadings, the same measurement errors, the same thresholds, and the same variances and covariances in the compared groups (male and female). All the restrictions were introduced step by step, checking the invariance.

The diagonally weighted least squares method was used, calculated on the polychoric correlation matrix (DWLS procedure). Finally, the following indices were used as goodness-of-fit measures (Hu & Bentler, 1999): Comparative Fit Index (CFI), Root-Mean-Square Error of Approximation (RMSEA), Standardized Root-Mean-Square Residual (SRMR), and Tucker-Lewis Index (TLI), where indices greater than 0.90 indicate good model fit, alongside root mean square error of approximation (RMSEA) values lower than 0.05, in addition to the Satorra-Bentler chi-square. To compare the goodness of fit between models, we use CFI (Cheung & Rensvold, 2002) and differences between chi-squares (Δχ^2^) we used criteria of -.01 for ΔCFI and.01 for ΔRMSEA.

Cronbach’s Alpha and McDonald’s Omega were used to estimate the reliability of the GQ-6/GQ-5 scores, and Spearman product moment correlation to check the evidence based on relations to other variables. Spearman’s correlation was used due to the non-fulfillment of the normality assumption for the questionnaire scores.

Jamovi 2.3.18 (The Jamovi Project, www.jamovi.org) was utilised for the exploratory and confirmatory factor analyses. Also, it was used for reliability indices. R studio 2022.07.2 (Posit Software, PBC) was used for all the remaining calculations.﻿

## Results

### Exploratory factor analysis

Considering the first split sample (*N* = 405), an Exploratory Factor Analysis was made with Principal Axis Factoring to extract factors with eigenvalues over one. First, a model with one factor and 6 items, second, a model eliminating item 6 (because of low factor loading less than.30, according to previous researchers; Brown,^2015^; Hair et al., 2019; Kline, 2015) (see Table 1). The Bartlett sphericity for the first model (6 items) was statistically significative [χ^2^ (15) = 527.05; *p* <.001], and the KMO was.74. The Bartlett sphericity second model (5 items) results also was statistically significative [χ^2^ (10) = 487.52; *p* <.001], and the KMO was.75.

### Confirmatory factor analysis

Considering the second split sample (*N* = 405), all indexes indicated that the 5-item model was the best fit to the data: RMSEA and SRMR are equal to or less than.08, and CFI and GFI (Goodness-of-Fit Index) are higher than .95 in all cases. Statistical differences were found between the model of 6 items and the model of 5 items (Δχ^2^ = 43.56, Δdf = 5; *p* <.05) (see Table 2).

**Table 2:** Confirmatory factor analysis (split sample: *N* = 405) and multigroup confirmatory factor analyses (diagonally weighted least squares; polychoric correlation matrices): male (n = 147) – female (*n* = 258)

Group/model	χ2*	df	RMSEA (CI 90%)	CFI	TLI	SRMR
Independent	1655.73	15				
GQ-6	50.27	9	.106 (.079–.135)	.968	.947	.059
GQ-5	6.71	4	.045 (.000–.073)	.995	.990	.024
GQ-5 Configural	10.25	8	.041(.000-.068)	996	991	.033
GQ-5 Metric	25.12	12	.050 (.000–.102)	.998	.997	.044
GQ-5 Scalar	30.50	22	.048 (.000–.076)	.999	.999	.039
GQ-5 Stric	30.25	23	.043 (.000–.072)	.999	.999	.040

^*^Satorra-Bentler’s chi-squared

The degree of relationship (standardized lambda weights) for each item with its corresponding factor in the 5-item model is shown in Table 3 (item 6 lambda =.21). The results show that all the items weigh on the factor to which they belong above.50 (see Table 3).

**Table 3:** Item number, item content, scale/item mean (M) and standard deviation (SD), item factor loading (lambda). split sample (*N* = 405)

Item	Item content	M	SD	Lambda (5 items)	Lambda (6 items)
1	I have so much in life to be thankful for [Tenho muito que agradecer na vida]	3.94	0.87	.91	.90
2	If I had to list everything that I felt grateful for, it would be a very long list [Elaboraria uma longa lista se tivesse que registar tudo pelo que estou agradecido(a)]	3.54	1.03	.89	.88
3	When I look at the world, I don’t see much to be grateful for [Quando olho para o mundo não vejo razões para exprimir muita gratidão]	3.37	1.10	.50	.53
4	I am grateful to a wide variety of people [Estou agradecido(a) a muitas pessoas]	3.74	0.90	.75	.75
5	As I get older I find myself more able to appreciate the people, events, and situations that have been part of my life history [À medida que fico mais velho(a) sou mais capaz de apreciar as pessoas, acontecimentos e situações que fizeram parte da minha história de vida]	4.03	0.88	.53	.54
6	Long amounts of time can go by before I feel grateful to something or someone [Eu demoro muito até me sentir grato a alguma coisa ou a alguém.]	4.03	0.88	-	.54

### Invariance testing

Moreover, all fit indices indicated that this model fit the data reasonably well for groups analysed. The CFI index of the baseline one-single group (i.e., total sample) equals the CFI indices of the multigroup analysis, allowing to conclude (Cheung & Rensvold, 2002) that gratitude scores showed total invariance (configurational, of factor loadings, of measurement errors, of intercepts, of variances and covariances) between male and female (using traditional criteria of -.01 for ΔCFI and .01 for ΔRMSEA) (see Table 2).

After establishing strict measurement invariance, we compared, in a post hoc analysis, the latent means across sex. Results indicated statistically significative higher means in the women group (ps < 0.01), the difference was small (βs: 0.14).

### Evidence based on relations to other variables and reliability of scores

Based on the results obtained in the CFA and as found in other studies, we decided to use the five-item version to carry out the examination of validity evidence based on relations to other variables, and the reliability of the scores. Means, standard deviations, Cronbach’s Alphas, McDonald’s Omegas, and correlations between GQ-5 score and other variables are shown in Table 4. The reliability indices are adequate, in according to Dunn et al.’s criteria (2014). Evidence based on relations to other variables is supported by the finding that GQ-5 score was positively correlated with satisfaction with life and negatively correlated with loneliness. Therefore, these results indicated that the more grateful the individual, the more likely the individual would present higher satisfaction with life, and lower loneliness.

**Table 4:** Means, Alphas and Omegas coefficients, and Spearman product moment correlation coefficients of GQ-5 with the variables (*N* = 362)

	M (SD)	Alpha	Omega	r Spearman
GQ-5	4.01 (0.78)	.77	.78	
Satisfaction with life	4.50 (1.16)	.84	.84	.47***
Loneliness	2.07 (0.63)	.82	.83	-.39***

^***^ *p* <.001

## Discussion

The main aim of this work was to validate a measure of gratitude—the Gratitude Questionnaire (GQ), which assesses dispositional gratitude (McCullough et al., 2002)—for the Portuguese cultural context. The findings provide evidence that the instrument functions adequately and yields scores that are reliable and support valid interpretations for the European Portuguese context and for the purposes of the present study. In this sense, the results support its appropriateness for assessing dispositional gratitude within this specific cultural and research setting, rather than making claims about universal validity. As noted earlier, the GQ has been used as a one-factor structure in prior studies. The present results align with this previous research, as they also support the unidimensionality of the measure.

The validity evidence among a Portuguese community population was evidenced. The findings of the current work showed (through EFA and CFA) that the one-factor five-item model of the Gratitude Questionnaire presented more adequate evidence of factorial structure than the original six-item model. Consistent with past works (Froh et al., 2011; Hudecek et al., 2020; Magallares et al., 2018; Rey et al., 2018; Yüksel and Oğuz Duran, 2012), item 6 (“Long amounts of time can go by before I feel grateful to something or someone”) was removed from the Portuguese GQ, as it displayed low factor loading in comparison with other GQ-6 items. In our study the decision to use the 5-item model rather than the original 6-item model for assessing evidence based on relations to other variables and the reliability of scores was grounded in the poor fit of the 6-item CFA and the consistently low factor loading of Item 6, a pattern also observed in several prior studies (e.g., Chen et al., 2009; Froh et al., 2011; Magallares et al., 2018; Rey et al., 2018). Given that internal consistency can increase simply by adding items—even when they contribute little conceptual or psychometric value—the inclusion of Item 6 would not provide additional information about gratitude. In line with this evidence, the European Portuguese version of the GQ was reduced to five items, and the resulting GQ-5 demonstrated good model fit and clear unidimensionality in the CFA, supporting its validity evidence. Dispositional gratitude, as assessed by the GQ-6, is conceptually understood to encompass four interrelated facets: frequency, intensity, span, and density (e.g., Cowles & Medvedev, 2024; McCullough et al., 2002). Although the original authors did not explicitly map individual items onto these facets, subsequent literature has interpreted the items according to their semantic content. Item 1 reflects the intensity of grateful feelings, whereas item 2 captures the span of gratitude by referring to the range of life domains that elicit grateful responses. Items 3 and 6, both reverse-scored, index the frequency with which gratitude is experienced, while item 4 represents density, as it refers to gratitude directed toward a wide variety of people. Item 5 reflects a combination of intensity and span, indicating both a deepening and a widening of gratitude experiences over time. Notably, even when item 6 is removed—as is the case in the GQ-5—none of the conceptual facets are lost, since all four facets remain represented across the remaining five items. Together, these items capture the multifaceted nature of dispositional gratitude while contributing to a unidimensional measurement structure.

As suggested by some researchers (e.g., Cabrera-Vélez et al., 2019; Chen et al., 2009; Dixit & Sinha, 2021), item 6 may have obtained a lower factor loading as it may be more difficult to understand. Plausible reasons for this increased difficulty include its higher level of abstraction, the inclusion of a temporal definition of gratitude that may make it difficult to understand, and the various interpretations that individuals may derive from the item’s reversed wording (Cabrera-Vélez et al., 2019; Chen et al., 2009; Dixit & Sinha, 2021).

Additionally, measurement invariance across sex was approached through multi-group CFA. According to previous studies (Garg et al., 2021; Hudecek et al., 2020; Llenares & Almeda, 2021), the findings supported that the one-dimensionality of GQ-5 was invariant across sex of the sample (for females and males).

Our results indicated that women experienced significantly higher grateful disposition than men. Prior studies also suggest that women are likely to be more grateful than men (e.g., Balgiu, 2020; Kashdan et al., 2009; Kong et al., 2015). Probably men may seek to avoid experiencing and expressing gratitude because of its association with vulnerability and weakness, “which may threaten their masculinity and social standing” (Kashdan et al., 2009, p. 692).

The reliability indices were similar to those reported in other validation studies with Cronbach’s Alpha coefficients between .70 and .80 (e.g., Balgiu, 2020; Chen et al., 2009; Rey et al., 2018). Thus, these reliability indices obtained can be considered adequate according to Dunn et al.’s (2014) criteria, indicating that the instrument provides sufficiently consistent GQ-5 scores for research purposes. Adequate reliability suggests that measurement error is maintained at a reasonable level, enabling the scale to discriminate meaningfully between individuals in their levels of dispositional gratitude. This level of internal consistency also supports the stability of the construct being assessed, increasing confidence that the observed scores reflect genuine differences rather than random variation.

Regarding evidence based on relations to other variables, the findings supported a significant association of gratitude with satisfaction with life in consonance with the theoretical framework and findings of previous studies (Balgiu, 2020; Chen et al., 2009; Lin, 2014; McCullough et al., 2002; Rey et al., 2018; Wood et al., 2008). By contrast, a negative association was evidenced with loneliness in consonance with the idea of the grateful disposition as “indispensable in the life of one individual who will face isolation and loneliness if the capacity to feel grateful is impaired” (Komter, 2004, p. 210). In sum, individuals evidencing a propensity for gratitude have been noted to present higher satisfaction with life, and lower loneliness.

There are several limitations to this study. First, we evaluated gratitude with a self-report measure that may introduce social desirability bias in the data. Future research should assess the social desirability (He et al., 2014). Secondly, the samples were composed of Portuguese adults of the general population, which limits the generalisability of the findings. Hence, further work is still required, considering, for example, adolescents and clinical patients. Thirdly, the cross-sectional design precludes causal inferences among the variables. Next research can use a longitudinal design to address this issue. Fourthly, our data were collected just in one country. Future research can examine measurement invariance of the GQ-5 across countries to test cross-cultural similarities and differences. Fifthly, this work has not considered the test–retest reliability of GQ-5 scores. Hence, next research should examine the test–retest of the GQ-5 in Portugal. Finally, the test has a small number of items and a single dimension, which could limit its sensitivity to capture fine nuances of the construct, but procedures such as factorial validation (EFA and CFA) and sample splitting using the Solomon method have been employed to minimize potential sample bias effects and ensure the robustness of the results.

## Conclusions

Despite these limitations, this work contributes to the literature on gratitude as it demonstrates that the use of the five-item instrument is appropriate for the measurement of the gratitude across the adult life span. Additionally, this study addresses the sex invariance of the instrument which is lacking in most of the revised literature. In conclusion, the results of the present research evidenced that the GQ-5 yields scores that are reliable and support valid interpretations for assessing the dispositional gratitude in both adult men and women in Portugal. Moreover, this study will help increase diagnostic accuracy, ensure comparability across groups, improve the test’s sensitivity, support more informed decisions and optimize future applications of the test. Hence, the GQ-5 might be utilized to evaluate gratitude in the Portuguese cultural context and can be possibly relevant for both research and professional practice in the future. Researchers could use the Portuguese GQ-5 to study antecedents, consequences, or correlates of the grateful disposition. On the other hand, considering the positive (e.g., satisfaction with life) and negative (e.g., loneliness) correlates of gratitude, among others, and the impact of the grateful experience on well-being (e.g., Froh et al., 2009; Portocarrero et al., 2020), the Portuguese GQ-5 might be used in professional practice.

## Data Availability

The data will be available in a public repository of the corresponding author’s university.

## Abbreviations

GQ-6: Gratitude Questionnaire – 6 items
GQ-5: Gratitude Questionnaire – 5 items (revised version without item 6)
GRAT: Gratitude Resentment and Appreciation Test
GAC: Gratitude Adjective Checklist
G-20: Gratitude Questionnaire – 20 items
MCGM: Multi-Component Gratitude Measure
SWLS: Satisfaction With Life Scale
ULS-6: UCLA Loneliness Scale – 6-item short form
RMSEA: Root Mean Square Error of Approximation
SRMR: Standardized Root Mean Square Residual
CFI: Comparative Fit Index
TLI: Tucker-Lewis Index
GFI: Goodness-of-Fit Index
Δχ^2^ (Delta Chi^2^): Change in Chi-square statistic (used for model comparison)
DWLS: Diagonally Weighted Least Squares
CFA: Confirmatory Factor Analysis
EFA: Exploratory Factor Analysis
SD: Standard Deviation
M: Mean
α (Alpha): Cronbach’s Alpha (reliability coefficient)
Ω (Omega): McDonald’s Omega (reliability coefficient)
